# Temporal trends and regional disparity in rheumatoid arthritis and gout hospitalizations in Sweden, 1998–2015

**DOI:** 10.1007/s10067-018-3983-8

**Published:** 2018-01-22

**Authors:** Aliasghar A. Kiadaliri, Martin Englund

**Affiliations:** 1 Lund University, Faculty of Medicine, Department of Clinical Sciences-Lund, Orthopaedics, Clinical Epidemiology Unit, Lund, Sweden; 2 Skåne University Hospital, Clinical Epidemiology Unit, Remissgatan 4, SE-221 85 Lund, Sweden; 3 Clinical Epidemiology Research and Training Unit, Boston University School of Medicine, Boston, MA USA

**Keywords:** Gout, Hospitalization, Regional disparity, Rheumatoid arthritis, Sweden, Temporal trend

## Abstract

Hospitalization is an important component of rheumatoid arthritis (RA) and gout economic burden. This study aimed to assess temporal trends and regional disparities in RA and gout hospitalizations among people aged ≥ 20 years in Sweden during 1998–2015. Data on hospital admissions with a principal diagnosis of RA or gout were collected from the National Patient Register. Age-standardized hospitalization rates (ASHRs) were calculated by means of direct standardization. The absolute and relative regional disparities were assessed. The temporal trends in ASHR and proportion of RA and gout hospitalizations from all and musculoskeletal disorders hospitalizations were analyzed using joinpoint regression. Between 1998–2000 and 2013–2015, the ASHR for RA declined by 78.9% from 109.9 to 23.2 per 100,000 Swedish adults, while it almost doubled for gout (from 10.5 to 20.8 per 100,000 Swedish adults). While in 1998–2000, RA hospitalizations were 10.3 times more frequent than gout (0.54 vs 0.05% of all hospitalizations), this ratio declined to 1.1 in 2013–2015 (0.13 vs 0.11% of all hospitalizations). The joinpoint regression revealed that, on average, the ASHR for RA declined by 10.2% (95% CI: 9.3–11.1) per year whereas for gout, it rose by 4.3% (3.2–5.4) per year during 1998–2015. While the relative regional disparities were stable, the absolute regional disparity declined for RA and increased for gout over the study period. While substantial decline in RA hospitalization is encouraging, the substantial rise in gout hospitalization is of concern, reflecting potential increase in prevalence of gout and suboptimal management of the disease.

**Electronic supplementary material:**

The online version of this article (10.1007/s10067-018-3983-8) contains supplementary material, which is available to authorized users.

## Introduction

Rheumatoid arthritis (RA) and gout are the two most common form of inflammatory arthritis associated with pain, functional impairment, reduced health-related quality of life, hospitalization, and increased mortality [1, 2]. In 2015, RA and gout were ranked as the 30th and 61st leading cause of years lived with disability in Sweden, respectively [3, 4]. In the recent decades, better health status, lower disease activity, increased remission rates, and improved survival for RA have been reported and attributed to improvement in the management of RA toward early and aggressive treatment with disease-modifying antirheumatic drugs [5–7]. These improvements led to reductions in orthopedic surgeries and hospitalizations for RA in several countries [8–11]. By contrast, studies reported persistent suboptimal management of gout [12, 13] with no improvement in premature mortality gap and rise in health care visits over recent decade [9, 10, 14–16]. Despite the rising burden of gout, to our knowledge, there is limited data on temporal trend in gout hospitalizations and its contrast with RA hospitalizations over recent decades. To address this, we estimated temporal trends in RA and gout hospitalizations and their regional disparities among people aged ≥ 20 years in Sweden from 1998 through 2015 using routinely collected data recorded in the Swedish National Patient Register.

## Method

### Data sources

We collected the public data from the National Board of Health and Welfare’s Patient Register (NPR) on hospitalizations among those aged 20 years and older in Sweden and across its 21 regions (http://www.socialstyrelsen.se/). These data include the principal diagnoses of almost all hospital admissions in Sweden by age, sex, region, and year from the year 1998 onward. The principal diagnoses are coded according to the International Classification of Diseases, the10th revision (ICD-10). We identified RA hospitalizations as diagnoses with the ICD-10 codes of M05-M06 and gout hospitalizations as diagnoses with the ICD-10 codes of M10. Furthermore, we identified hospitalizations of musculoskeletal (MSK) disorders as diagnoses with the ICD-10 codes of M00–M99. We obtained the data on population by sex, age, region, and year from the Statistics Sweden (http://www.scb.se).

### Temporal trend analysis

We used joinpoint regression to assess the temporal trends in three measures: age-standardized hospitalization rates calculated by means of direct standardization using the Sweden population in the year 2000 as standard, proportions of RA and gout hospitalizations from MSK disorders hospitalizations, and proportions of RA and gout hospitalizations from all hospitalizations. We used the Joinpoint Regression Program version 4.2.0.2 from the Surveillance Research Program of the US National Cancer Institute (http://surveillance.cancer.gov/joinpoint). This program applies a series of permutation tests to compute the number of joinpoints to best fit the data and estimate an annual percentage change (APC) for each joinpoint. To provide a summary measure of the trend for the whole time period, the average annual percent change (AAPC) is calculated as the weighted average of APCs. We also assessed the temporal trend in the average length of hospital stay using the non-parametric Mann-Kendall trend test [17].

### Regional disparity

We assessed the absolute regional disparities by calculating the absolute weighted mean difference from overall mean as the sum of the absolute difference in each region’s age-standardized hospitalization rate from the overall rate for whole country, weighted by the region’s proportion of the Sweden population. We measured the relative regional disparities using the index of disparity, calculated as the average of the absolute differences between age-standardized hospitalization rate in each region and the overall rate, divided by the overall rate and expressed as a percentage [18]. The temporal trend in the absolute and relative regional disparities was evaluated using the Mann-Kendall trend test.

## Results

### Hospitalizations

**Fig. 1:**
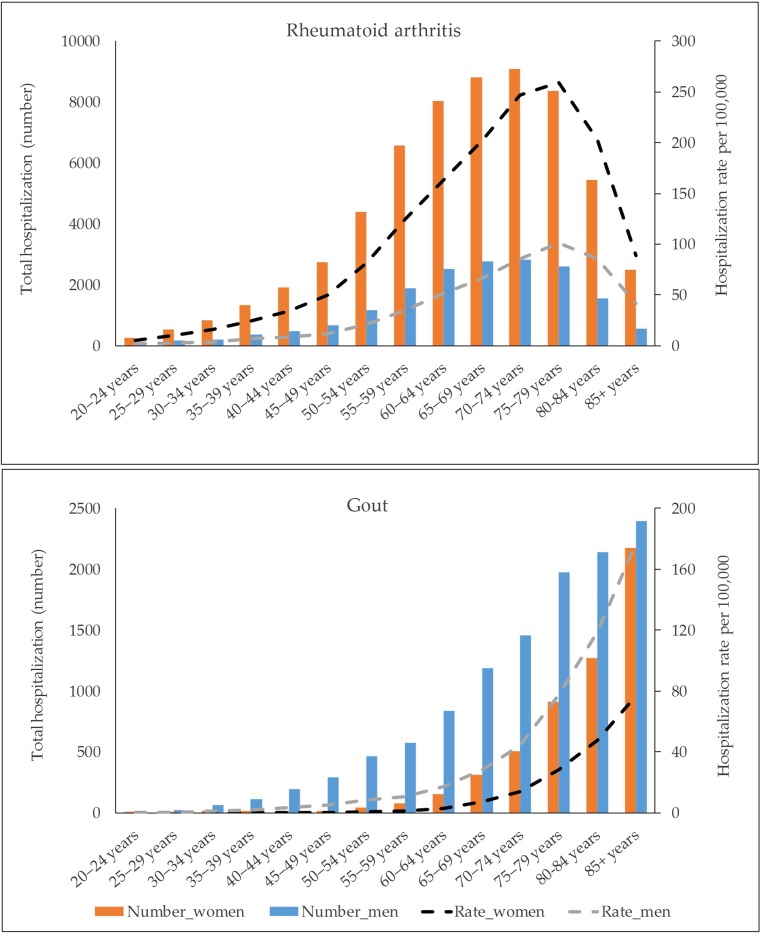
The absolute number and mean age-specific rate per 100,000 Swedish adults for rheumatoid arthritis and gout hospitalizations, 1998–2015

There were 78,689 RA and 17,283 gout hospitalizations between 1998 and 2015. About 77% of RA hospitalizations were for women and 57% among persons 65 years and older. Corresponding figures for gout hospitalizations were 32 and 83%, respectively. RA hospitalization rate rose up to 80 years and declined thereafter, whereas gout hospitalization rate consistently rose with age (Fig. 1). The proportions of RA from MSK disorders and all hospitalizations were greater than gout for all age groups except those aged ≥ 85 years (Fig. S1 in supplement).

### Temporal trends

Between 1998–2000 and 2013–2015, the mean age-standardized rates for RA declined by 78.9% (95% CI 78.5, 79.4) from 109.9 (108.4, 111.4) to 23.2 (22.5, 23.8) per 100,000 Swedish adults, whereas for gout, it rose by 98.2% (88.2, 108.9) from 10.5 (10.0, 11.0) to 20.8 (20.2, 21.4) per 100,000 Swedish adults. While in 1998–2000 RA hospitalizations were 10.3 times more frequent than gout (0.54 vs 0.05% of all hospitalizations), this ratio declined to 1.1 in 2013–2015 (0.13 vs 0.11% of all hospitalizations).

**Fig. 2:**
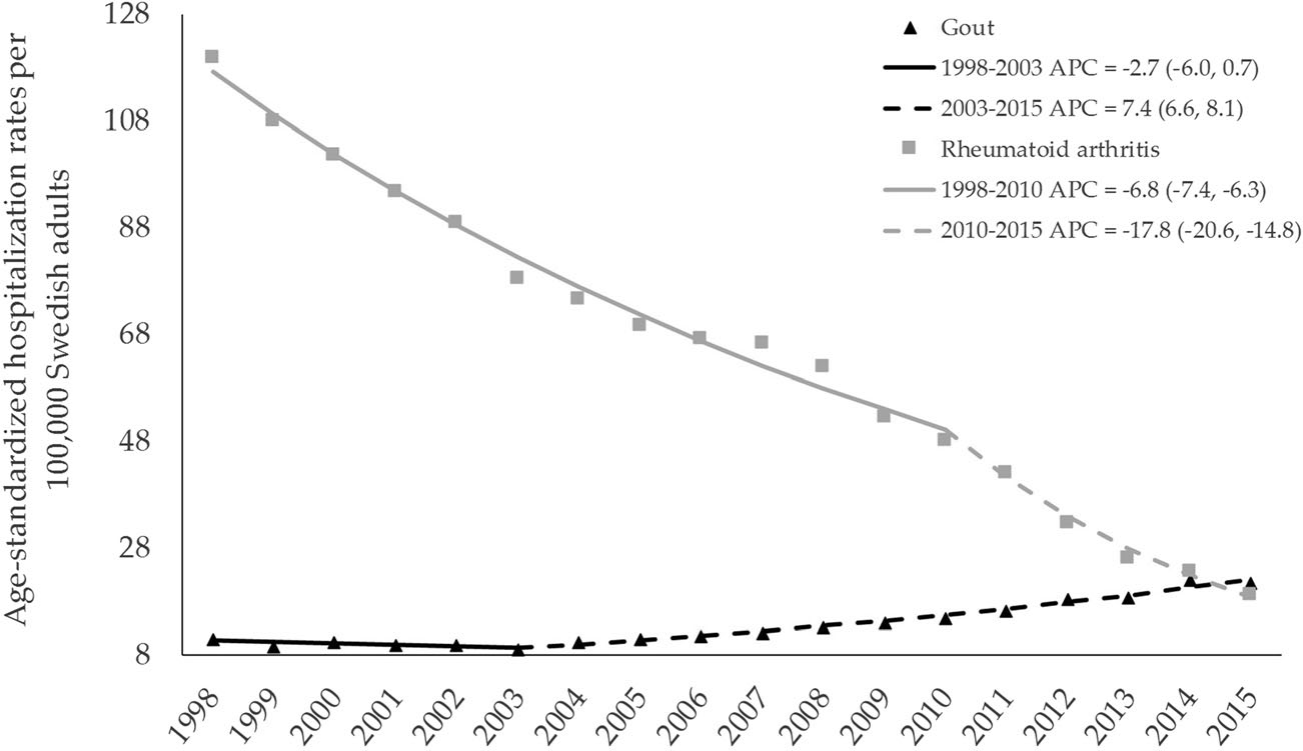
Annual age-standardized hospitalization rates per 100,000 Swedish adults for rheumatoid arthritis and gout, 1998–2015. Symbols display the observed values and solid lines indicate fitted values using joinpoint regression. For each joinpoint, the annual percentage change (APC) and its 95% confidence interval are reported

Joinpoint regression suggested that the annual age-standardized hospitalization rate for RA declined by 6.8% (95% CI 6.3, 7.4) per year from 1998 through 2010 and declined by 17.8% (14.8, 20.6) per year thereafter (Fig. 2), resulting in an average annual reduction of 10.2% (9.3, 11.1) for the whole study period (Table 1). In contrast, the annual age-standardized hospitalization rate for gout was stable from 1998 to 2003 and rose by 7.4% (6.6, 8.1) per year thereafter, resulting in an average annual increase of 4.3% (3.2, 5.4) for the whole study period. These reverse trends led to a higher age-standardized hospitalization rate for gout compared to RA in 2015 (among men, this occurred in 2010 and continued henceforth, Fig. S2 in supplement). The similar trends (i.e., reduction for RA and rise for gout) were observed for the proportions of RA and gout hospitalizations from MSK disorders and all hospitalizations. These trends persisted across sex and age subgroups (Fig. S2 and Fig. S3 in supplement). While the average length of hospital stay for RA declined in both sexes (Z = − 4.92 in men and Z = − 5.0 in women, *P* < 0.001 in both sexes), there were no statistically significant trends for gout (Z = − 0.64, *P* = 0.52 in men and Z = − 1.59, *P* = 0.11 in women).

**Table 1:** Temporal trends in rheumatoid arthritis and gout hospitalizations in Sweden by sex and age, 1998–2015

	% change (95% CI) between 1998–2000 and 2013–2015	Average annual percent change (95% CI), 1998–2015		
	Rate^a^	Rate^a^	Proportion from all hospitalizations	Proportion from MSK disorders hospitalizations
	Rheumatoid arthritis			
All	− 78.9 (− 79.4, − 78.5)	− 10.2 (− 11.1, − 9.3)	− 9.2 (− 9.8, − 8.5)	− 10.2 (− 11.1, − 9.3)
Men	− 80.5 (− 81.7, − 79.2)	− 10.3 (− 11.8, − 8.9)	− 9.3 (− 10.4, − 8.3)	− 10.8 (− 12.1, − 9.5)
Women	− 78.1 (− 78.8, − 77.3)	− 9.7 (− 10.5, − 9.0)	− 9.1 (− 9.8, − 8.4)	− 9.7 (− 10.7, − 8.8)
Age groups				
20–49 years	− 78.2 (− 80.1, − 76.2)	− 8.8 (− 10.4, − 7.2)	− 8.8 (− 10.3, − 7.3)	− 8.0 (− 9.6, − 6.5)
50–64 years	− 82.2 (− 83.3, − 81.1)	− 11.4 (− 12.7, − 10.0)	− 10.2 (− 11.3, − 9.2)	− 11.6 (− 12.7, − 10.4)
65–79 years	− 79.3 (− 80.1, − 78.3)	− 10.1 (− 11.3, − 8.8)	− 8.6 (− 9.6, − 7.7)	− 10.1 (− 11.1, − 9.0)
80+ years	− 70.0 (− 72.2, − 67.5)	− 7.7 (− 8.8, − 6.5)	− 8.0 (− 9.0, − 6.9)	− 8.8 (− 9.9, − 7.7)
	Gout			
All	98.2 (88.2, 108.9)	4.3 (3.2, 5.4)	5.3 (3.9, 6.8)	4.7 (3.7, 5.7)
Men	107.2 (94.2, 121.0)	4.7 (3.3, 6.2)	5.8 (4.4, 7.2)	4.9 (3.6, 6.1)
Women	69.5 (55.0, 85.2)	3.1 (1.5, 4.6)	4.5 (3.2, 5.9)	3.8 (2.6, 5.0)
Age groups				
20–49 years	39.1 (9.5, 76.8)	2.8 (1.0, 4.7)	2.7 (1.1, 4.3)	3.6 (2.0, 5.3)
50–64 years	103.6 (75.3, 136.6)	4.7 (1.2, 8.3)	7.0 (5.6, 8.5)	5.0 (3.6, 6.5)
65–79 years	83.2 (68.4, 99.3)	4.6 (3.3, 5.9)	6.0 (4.8, 7.1)	4.2 (3.2, 5.2)
80+ years	111.5 (95.6, 128.7)	4.6 (2.8, 6.5)	4.8 (2.6, 7.1)	3.8 (2.1, 5.5)

All changes are statistically significant (*p* < 0.05)

*CI* confidence interval, *MSK* musculoskeletal

^a^In the joinpoint regression analysis, we used age-standardized rates for overall and sex-stratified analyses and age-specific rates for age-stratified analyses

### Regional disparities

For RA, there were 2.9-fold (95% CI: 2.6, 3.3) difference between the regions with the highest and lowest mean age-standardized hospitalization rates. The corresponding figure for gout was 2.1 (1.7, 2.5). The absolute regional disparities measured by the absolute weighted mean difference from overall mean ranged from 3.9 to 28.4 per 100,000 person-years for RA and from 1.4 to 6.5 per 100,000 person-years for gout (Table S1 in supplement). Between 1998 and 2015, the absolute disparity for RA declined (Z = − 4.1, *P* < 0.001) and that for gout increased (Z = + 3.9, P < 0.001). The index of disparity ranged from 18.5 to 35.7% for RA and from 16.6 to 41.2% for gout. There were no statistically significant changes in the index of disparity over the study period (Z = − 0.8, *P* = 0.41 for RA and Z = − 0.5, *P* = 0.60 for gout).

## Discussion

We found substantial decline in the absolute and relative burden of RA hospitalizations in Sweden during the recent two decades, whereas those for gout almost doubled at the same time. Due to these opposite trends, age-standardized hospitalization rate of gout surpassed the rate for RA in 2015. While there were statistically significant changes in the absolute regional disparity (declined for RA and rose for gout), there were no statistically significant trends in the relative regional disparities.

The declining trend in RA hospitalizations in our study is consistent with decreasing trends in orthopedic surgeries and hospitalizations for RA reported from different locations [8–11, 19]. These reductions are mainly attributed to better management of RA including treating to target, early and more aggressive use of disease-modifying antirheumatic drugs, and introduction of biologic agents [6, 9]. Although reductions in RA hospitalizations and hospital days imply decrease in RA hospitalization costs, this decrease likely indicates a shift in RA costs rather than reduction in RA total costs (i.e., reduction in hospitalization costs offset by rise in medication costs) [6, 11, 19]. A previous study in Sweden reported that substantial decline in hospital admissions and hospital days for RA from 1990 to 2010 resulted in a marked reduction in share of hospitalization costs from total RA costs (from 15% in 1990 to 3% in 2010) [11]. On the other hand, RA outpatient physician visits remained relatively unchanged and inflation-adjusted total costs for RA rose by 32% over the same period mainly due to the introduction of biologic drugs [11].

In contrast to the decline in RA hospitalizations, our results showed that hospitalization rates for gout almost doubled over the study period. The rising trends in gout hospitalizations have been reported in several studies [9, 10, 16, 20]. The increasing trend in gout hospitalizations in our study is consistent with marked increase in gout incidence in Sweden over the recent decade [12]. While the role of obesity epidemic, increasing use of diuretics and aspirin, shifts in diet and lifestyle, and rising gout awareness should not be overlooked, the substantial rises in gout hospitalizations very likely reflect persistent suboptimal gout management, medication non-compliance, and poor adherence to current gout guidelines [13, 20–23]. Two recent studies from western Sweden found that only a minority of gout patients receive urate lowering therapy (e.g., only 25% of hospitalized gout patients were on recommended urate lowering therapy preceding admission [20, 23]). Considering that gout is a treatable disease, this suboptimal care implies that many of gout hospitalizations are potentially preventable (e.g., a study noted that 89% of hospitalizations with a primary diagnosis of gout were preventable [24]). The observed rise in gout hospitalizations with no statistically significant changes in the average length of hospital stay have very likely led to rise in hospitalization costs for gout. Previous studies in Canada [9] and USA [10] suggested that doubled hospitalization rate for gout was associated with 125 and 68% rises in inflation-adjusted gout hospitalization costs per 100,000 people over time. A recent study in Western Sweden reported that hospitalization rate and inflation-adjusted total annual hospitalization costs for gout increased by 45 and 56%, respectively, between 2009 and 2012 [20].

The regional disparities in RA and gout hospitalizations in our study are consistent with geographic variations in incidence, prevalence, and treatment of these conditions [13, 25]. In addition, regional differences in clinical practice including adherence to treatment guidelines, in environmental exposures, in distribution of risk factors including lifestyle, in socioeconomic factors, and in cultural norms including attitudes toward sickness benefits might partially explain the regional disparities in RA and gout hospitalizations [13, 25]. Furthermore, despite the presence of the national health insurance with universal access to health care in Sweden, possibility of regional differences in availability of rheumatology specialists, and in patients’ health-seeking behavior and health care utilization cannot be ruled out.

Simultaneous investigation of the two most common form of inflammatory arthritis, the use of national inpatient data spanned over about two decades, and assessing both absolute and relative burden of hospitalizations are the main strengths of our study. However, several limitations of our study should be acknowledged. Misdiagnosis and coding errors in administrative data, particularly potential variations by time and place, is of concern. While in overall the principal diagnosis for about 1% of hospital admissions in the NPR is missing, potential regional variation in missingness is another source of concern. Due to substantial variations in coverage and quality over time, we did not include specialized outpatient care (including day surgeries) and primary care visits. For example, while the data on specialized outpatient care are available since 2001, the proportion of specialized outpatient care visits with missing primary diagnosis dropped from 25 to 30% in early years to about 4% in 2016 (http://www.socialstyrelsen.se) that complicates conducting a proper temporal trend analysis. This implies that only those patients with more severe conditions requiring hospital admission are included in the study and hence generalizability of the findings to less severe health care visits is limited.

## Conclusion

There were favorable reductions in the absolute and relative burden of RA hospitalization in Sweden over the study period. However, hospitalizations for gout substantially rose highlighting the need for reassessment of gout management.

## Electronic supplementary material

ESM 1 (PDF 1313 kb)
