# Supplementary material for: Temporal trends and regional disparity in rheumatoid arthritis and gout hospitalizations in Sweden, 1998–2015
Source: Clin Rheumatol. 2018 Jan 22;37(3):825–30. doi: 10.1007/s10067-018-3983-8 (PMC5835057; doi:10.1007/s10067-018-3983-8)

Table S1. The absolute and relative disparities for rheumatoid arthritis (RA) and gout hospitalizations in Sweden, 1998-2015.

	1998	1999	2000	2001	2002	2003	2004	2005	2006	2007	2008	2009	2010	2011	2012	2013	2014	2015	Z score	P for trend
	Absolute weighted mean difference from overall mean per 100,000 persons																			
RA	25.8	28.4	25.5	26.8	19.9	13.4	17.6	19.4	20.8	23.6	20.3	11.6	12.3	10.1	7.2	4.4	3.9	4.7	**-4.1**	<0.001
Gout	1.4	1.8	2.9	3.2	2.2	2.3	3.4	1.9	2.3	2.7	3.1	3.5	2.6	3.7	4.0	5.0	6.3	6.5	**+3.9**	<0.001
	Index of disparity (%)																			
RA	25.3	28.6	25.1	28.1	28.8	21.9	27.5	34.9	33.3	35.7	34.9	26.4	30.2	27.1	22.1	19.8	18.5	27.5	-0.8	0.41
Gout	16.6	22.4	31.9	35.5	41.2	29.2	34.4	17.2	26.8	27.6	23.2	23.9	18.8	21.9	22.1	26.9	25.3	28.8	-0.5	0.60

*Statistically significant results (p<0.05) are in bold.

Figure S1. Proportions of rheumatoid arthritis and gout hospitalizations from (a) musculoskeletal disorders hospitalizations, and (b) all hospitalizations by age groups, 1998-2015.

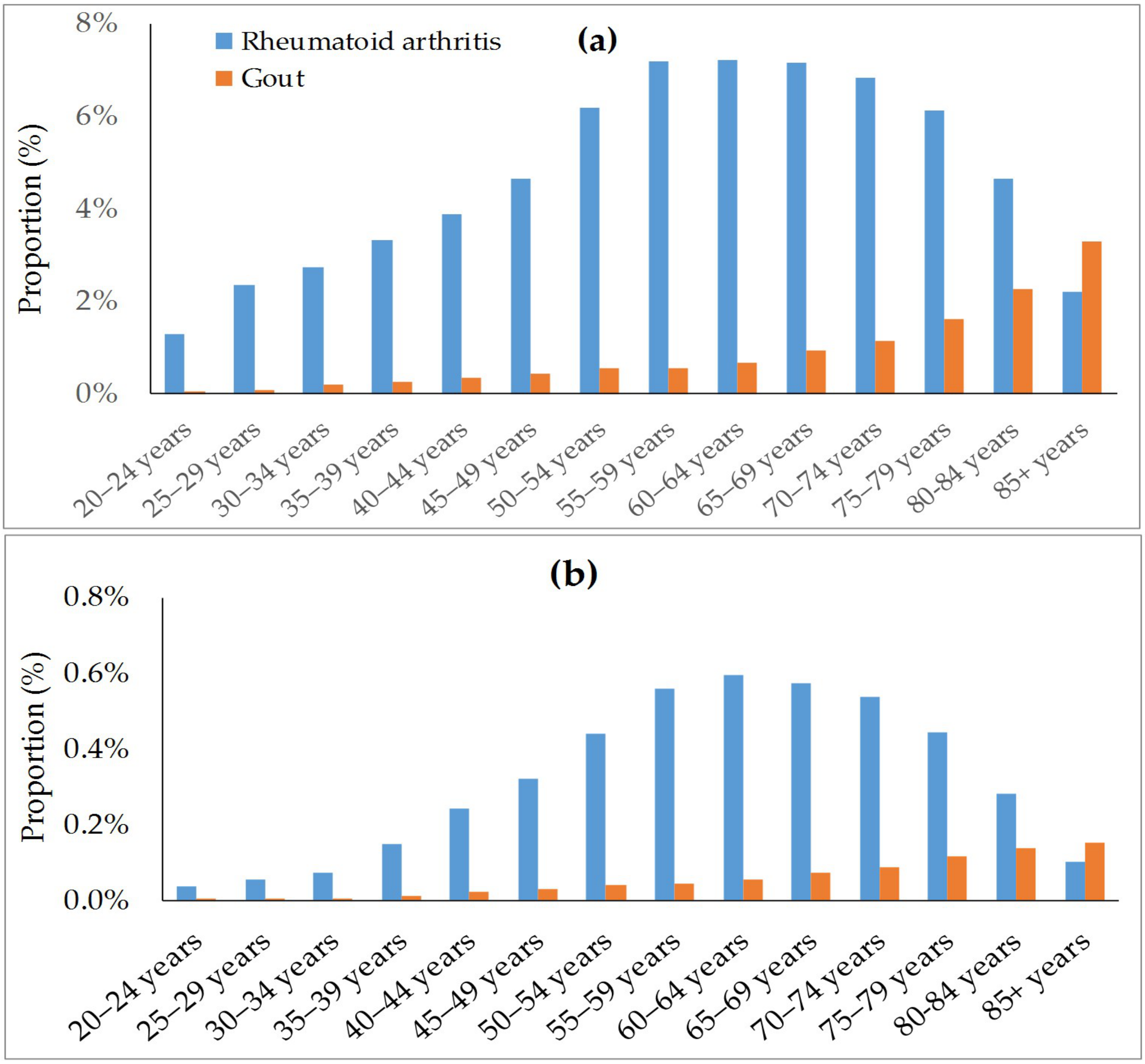

Figure S2. Annual age-standardized hospitalization rates per 100,000 Swedish adults for rheumatoid arthritis and gout by sex, 1998-2015. Symbols display the observed values and solid lines indicate fitted values using joinpoint regression. For each joinpoint, the annual percentage change (APC) and its 95% confidence interval are reported.

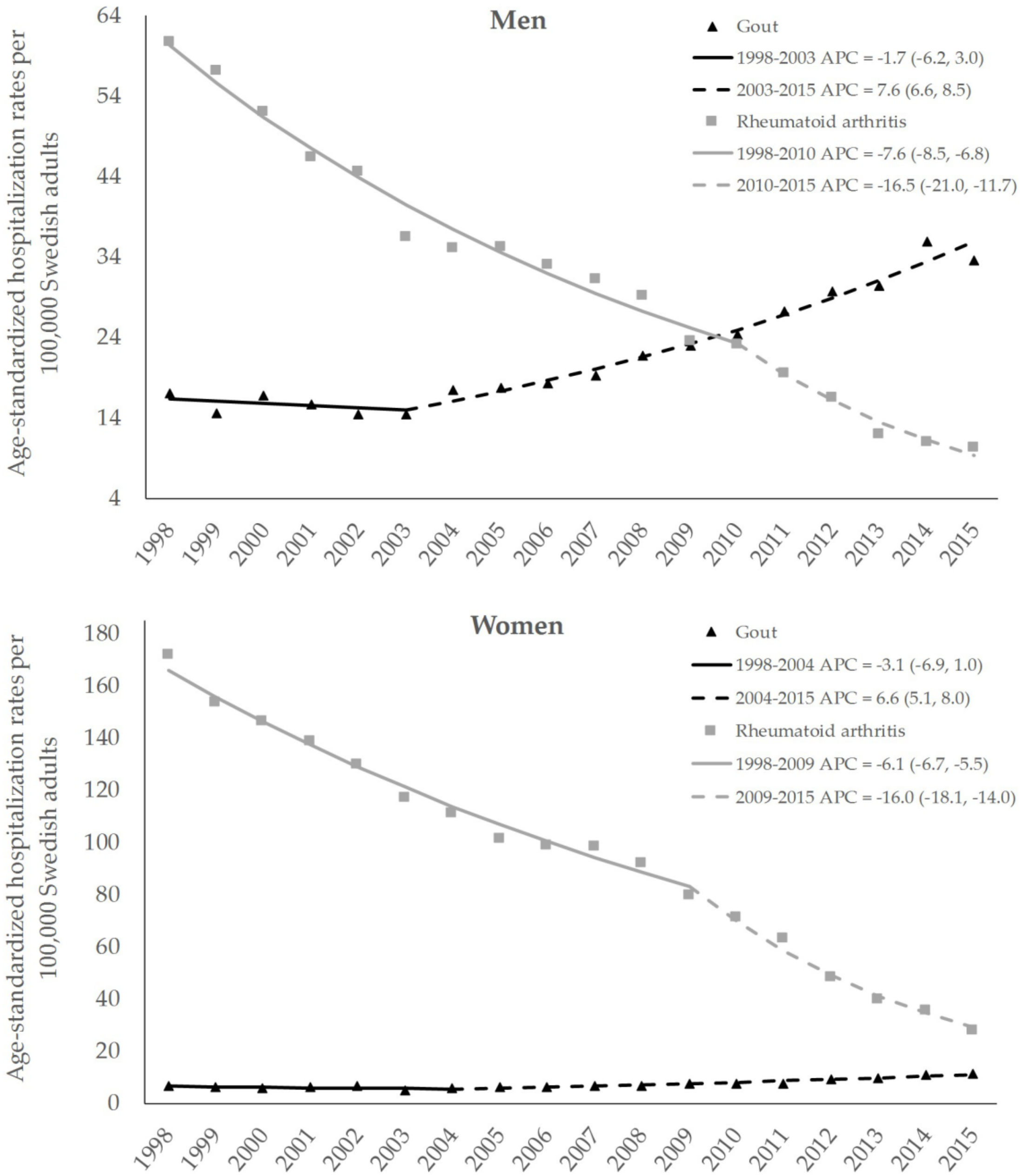

Figure S3. Annual hospitalization rates per 100,000 persons for rheumatoid arthritis and gout by age groups, 1998-2015. Symbols display the observed values and solid lines indicate fitted values using joinpoint regression. For each joinpoint, the annual percentage change (APC) and its 95% confidence interval are reported.

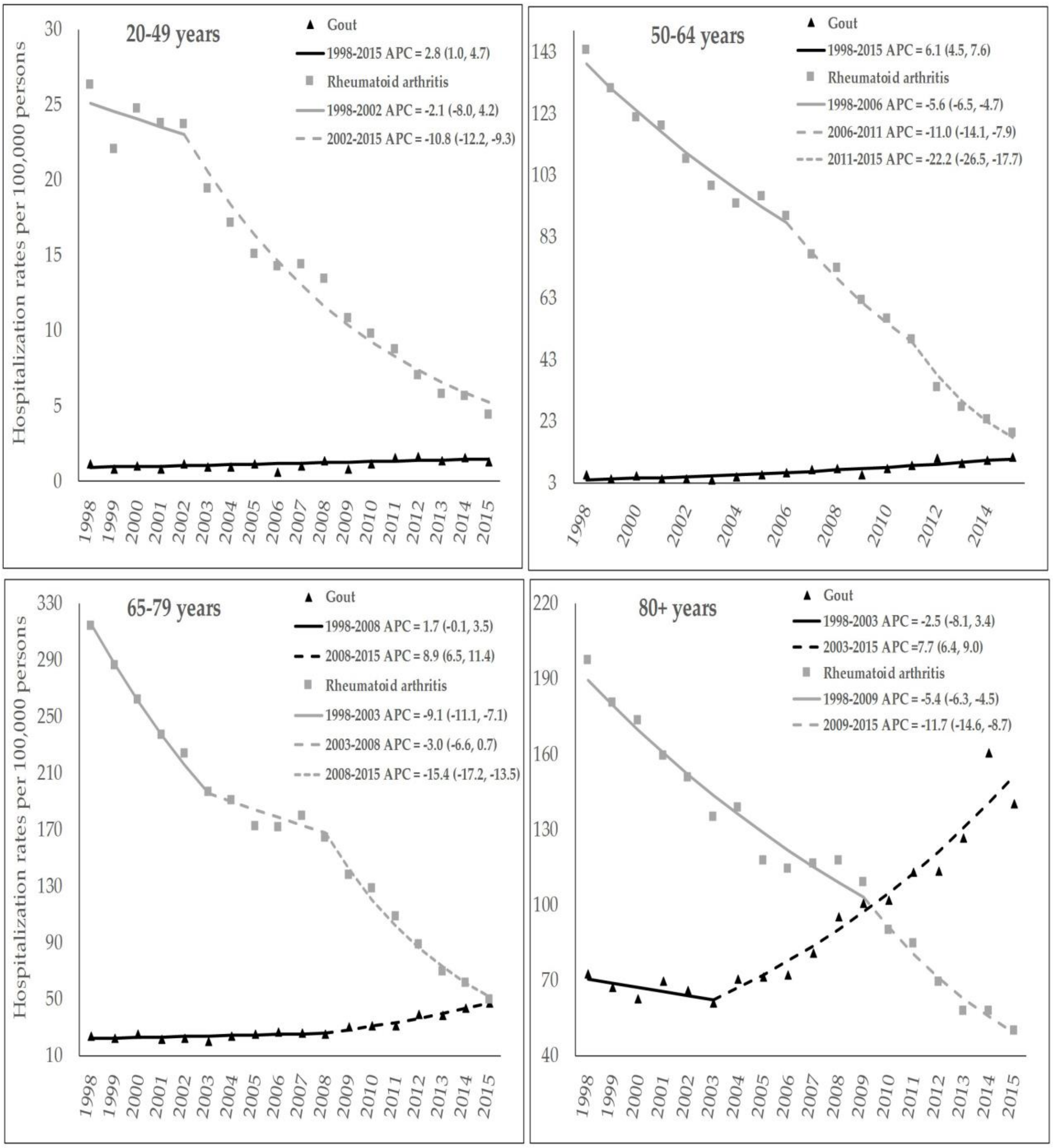

Supplement: Supplementary file 1 — (PDF 1313 kb) [file 10067_2018_3983_MOESM1_ESM.pdf]
